# Understanding How Staphylococcal Autolysin Domains Interact With Polystyrene Surfaces

**DOI:** 10.3389/fmicb.2021.658373

**Published:** 2021-05-19

**Authors:** Radha P. Somarathne, Emily R. Chappell, Y. Randika Perera, Rahul Yadav, Joo Youn Park, Nicholas C. Fitzkee

**Affiliations:** ^1^Department of Chemistry, Mississippi State University, Mississippi State, MS, United States; ^2^Department of Biochemistry, Chemistry, and Center for Structural Biology, Vanderbilt University, Nashville, TN, United States; ^3^Department of Comparative Biomedical Sciences, College of Veterinary Medicine, Mississippi State University, Mississippi State, MS, United States

**Keywords:** biofilms, autolysin proteins, polystyrene nanoparticles, medical implants, surface chemistry

## Abstract

Biofilms, when formed on medical devices, can cause malfunctions and reduce the efficiency of these devices, thus complicating treatments and serving as a source of infection. The autolysin protein of *Staphylococcus epidermidis* contributes to its biofilm forming ability, especially on polystyrene surfaces. R2ab and amidase are autolysin protein domains thought to have high affinity to polystyrene surfaces, and they are involved in initial bacterial attachment in *S. epidermidis* biofilm formation. However, the structural details of R2ab and amidase binding to surfaces are poorly understood. In this study, we have investigated how R2ab and amidase influence biofilm formation on polystyrene surfaces. We have also studied how these proteins interact with polystyrene nanoparticles (PSNPs) using biophysical techniques. Pretreating polystyrene plates with R2ab and amidase domains inhibits biofilm growth relative to a control protein, indicating that these domains bind tightly to polystyrene surfaces and can block bacterial attachment. Correspondingly, we find that both domains interact strongly with anionic, carboxylate-functionalized as well as neutral, non-functionalized PSNPs, suggesting a similar binding interaction for nanoparticles and macroscopic surfaces. Both anionic and neutral PSNPs induce changes to the secondary structure of both R2ab and amidase as monitored by circular dichroism (CD) spectroscopy. These changes are very similar, though not identical, for both types of PSNPs, suggesting that carboxylate functionalization is only a small perturbation for R2ab and amidase binding. This structural change is also seen in limited proteolysis experiments, which exhibit substantial differences for both proteins when in the presence of carboxylate PSNPs. Overall, our results demonstrate that the R2ab and amidase domains strongly favor adsorption to polystyrene surfaces, and that surface adsorption destabilizes the secondary structure of these domains. Bacterial attachment to polystyrene surfaces during the initial phases of biofilm formation, therefore, may be mediated by aromatic residues, since these residues are known to drive adsorption to PSNPs. Together, these experiments can be used to develop new strategies for biofilm eradication, ensuring the proper long-lived functioning of medical devices.

## Introduction

*Staphylococcus epidermidis* is a very common pathogen known to cause a variety of infections, including those involving medical implants and devices (McCann et al., 2008; Büttner et al., 2015; Zheng et al., 2018). The cell wall of *S. epidermidis* contains protein, nucleic acid, and peptidoglycan components (Büttner et al., 2015), and a major protein component of the cell wall is the peptidoglycan hydrolase, autolysin (AtlE). The AtlE protein plays an essential role in bacterial cell wall cleavage and is therefore critical during cell division (Nega et al., 2020). The R2ab and amidase domains in AtlE are responsible for enzyme targeting in the septum region (Yamada et al., 1996). AtlE is post-translationally cleaved between the R2 and R3 domains, resulting in a construct containing the amidase enzyme and the R1 and R2 repeats (Amidase-R1-R2). The protein is non-covalently associated with the cell wall through interactions between the R1 and R2 repeats and lipoteichoic acid (LTA) (Zoll et al., 2012). The native structures of both R2ab and amidase have been solved by Zoll et al. (2010, 2012) but the structure of the LTA-complexed protein is not currently known. In addition, AtlE is known to take part in its primary attachment to surfaces, especially to polystyrene surfaces (Houston et al., 2011). Both amidase and R2ab are required for *S. epidermidis* to be biofilm positive on polystyrene surfaces (Heilmann et al., 1997; Zoll et al., 2012).

Biofilms are assemblies of microbial cells present in a highly structured community, with the ability to attach to surfaces and form colonies that are associated with a extracellular polymeric matrix (Sandal et al., 2007). Biofilm formation occurs in three steps – attachment, maturation and dispersion (Crouzet et al., 2014). In the attachment stage, microorganisms adhere on to non-biological surfaces by non-specific hydrophobic interactions (Dunne, 2002). Biofilms have many unfavorable effects on public health, especially since they infect medical implants in the body such as heart valves and catheters (Trautner and Darouiche, 2004). In fact, they account for more than half of microbial infections (Jamal et al., 2018). They can also form on a variety of biological surfaces such as the urinary tract, skin, and most commonly on teeth (Saini et al., 2011). *S. epidermidis*, being an opportunistic pathogen, can attach onto polymeric devices and cause harm to both healthy and immunocompromised patients. There are three basic components that are basic, vital components in biofilm formation – microbes, extracellular matrix, and a surface (Donlan, 2002). If any of these components are removed, biofilm formation would not happen. However, biofilm formation is a fairly complex process that cannot be restricted to merely these three main components. Many other factors such as the type of organism, surface, genetic variations, and other environmental factors serve a purpose in biofilm formation (Steenackers et al., 2016; Ponomareva et al., 2018).

Preventing biofilm formation is imperative for the optimum function of biomedical implants and diagnostic equipment. Polystyrene is an important surface for these applications, because it is abundant in the medical field, and because it is amenable to surface modification. While polystyrene is not currently used in implantable medical devices (Sastri, 2010), it is a common component of medical diagnostic equipment (Loos et al., 2014), and biofilms are observed to form readily on untreated polystyrene surfaces (Kaplan et al., 2004). These biofilms can lead to complications associated with device failure (Gominet et al., 2017). Primary adhesion of bacteria to abiotic surfaces is usually facilitated by non-specific interactions. Polystyrene is amenable to surface conditioning, an approach that can be used to alter these interactions, reducing biofilm formation through surface pretreatment (Lorite et al., 2011). However, conditioning remains challenging because it requires an understanding of the molecular interactions involved in surface adhesion. Some conditioners tend to hinder biofilm growth by killing bacterial cells (Zhang et al., 2013; Percival et al., 2014), emphasizing the importance of understanding adsorption to abiotic surfaces. Therefore, it is of high interest to understand how molecules interact with polystyrene surfaces: not only is this knowledge relevant for understanding biofilm formation on many medical diagnostic tools, it is also important for preventing biofilms through surface conditioning.

In this study, we explore the behavior of the R2ab and amidase domains on polystyrene surfaces. In addition to examining these proteins’ behavior with macroscopic surfaces, such as cell plates, we explore how these protein domains interact with carboxylate functionalized, polystyrene nanoparticles (PSNPs). Nanoparticles have a large surface-to-volume ratio, and they can be functionalized to remain suspended in solution at high concentrations (Fang et al., 2009). Moreover, nanoparticles of diameter 15 nm and larger are several orders of magnitude larger than small protein domains like R2ab and amidase, making them a fair approximation for a flat surface. Our group (Wang et al., 2014; Woods et al., 2016), and others (Lacerda et al., 2010; Gunawan et al., 2014; Satzer et al., 2016) have found that protein binding occurs independently of nanoparticle curvature for nanoparticles larger than 15 nm. However, surface functionalization and protein identity can lead to curvature-dependent effects (Gagner et al., 2011; Perera et al., 2019; Visalakshan et al., 2020), and this remains an area of active investigation. By studying both R2ab and amidase on a nanoparticles and flat surfaces, we aim to understand the structural behavior of these proteins when adsorbed to surfaces and to understand the broader principles of protein-surface interactions. Ultimately, we have analyzed the interaction of the autolysin proteins R2ab and amidase with both polystyrene surfaces and nanoparticles to achieve a better understanding of these interactions and how they compare. In the long term, this could lead to novel potential solutions for eradicating biofilms formed on medical devices.

## Materials and Methods

### Protein Expression and Purification

Plasmids encoding the sequence for R2ab and amidase domains were synthesized by Life Technologies, Inc. (Carlsbad, CA, United States) and transformed into a pET-15b vector; these sequences were identical to the sequences used previously (Zoll et al., 2010, 2012; Büttner et al., 2014). Both vectors were transformed into *Escherichia coli* BL21(DE3) cell. Transformed cells containing the R2ab plasmid were grown overnight at 37°C for 16 h in LB media. This starter culture was then used to inoculate 1L of LB media, at an initial OD_600_ of 0.05. When this larger culture reached an OD_600_ of 0.5–0.7, it was induced using 0.5 mM isopropyl β-d-1-thiogalactopyranoside (IPTG), and the culture was allowed to incubate overnight at 25°C. The cells were then harvested by spinning them for 30 min at 8,000 × *g* and the pellets were resuspended in lysis buffer (150 mM NaCl, 20 mM HEPES pH 7.5, 40 mM imidazole). The resuspended cells were sonicated at 45% power, thrice with 2 min continuous pulsing, followed by 2 min rest, for a total process time of 6 min. The resulting lysate was centrifuged at 32,000 × *g* for 30 min at 4°C. R2ab was collected from the soluble fraction and purified through a 5 mL Nickel HisTrap FF column (Cytiva Life Sciences, Marlborough, MA, United States) that was equilibrated with lysis buffer. The bound protein was eluted using a gradient of elution buffer (150 mM NaCl, 20 mM HEPES pH 7.5, 800 mM Imidazole). Thrombin was added to the protein after quantification to remove the histidine tag, and then dialyzed in dialysis buffer (150 mM NaCl, 20 mM HEPES pH 7.5, 20 mM Imidazole) overnight. Thrombin was removed from the protein using benzamidine beads (Cytiva Life Sciences). After centrifuging and filtering the beads, the supernatant was run once more through a 5 mL Nickel HisTrap FF column to obtain tag-free protein. Finally, the purified protein was run through a Superdex 26/600 75 column, equilibrated using gel filtration buffer (50 mM NaCl, 20 mM sodium phosphate pH 6.5). Purified R2ab protein was lyophilized and stored at −80°C. Purification of amidase was performed similarly, except that the lysis buffer used, contained 50 mM sodium phosphate, pH 7.4, 300 mM NaCl and 25 mM imidazole, and the protein was eluted using 50 mM sodium phosphate, pH 7.4, 300 mM NaCl 400 mM imidazole.

### Nanoparticle Preparation and Characterization

Carboxyl-functionalized polystyrene nanoparticles with a nominal diameter of 20 nm were purchased from Thermo Fisher (catalog #C37261, lot #1688129; Eugene, OR, United States). The manufacturer-listed nanoparticle diameter is 28 ± 6 nm by transmission electron microscopy (TEM); this was confirmed by dynamic light scattering (DLS) using an Anton Paar Litesizer 500, where the hydrodynamic diameter was observed to be 40 nm. The stock solution concentration was 4% (w/v) total solids. The number of carboxyl groups per nanoparticle (59 COOH groups per particle), determined by titration, was taken from the manufacturer’s certificate of analysis for this lot. Neutral, non-functionalized polystyrene nanospheres were obtained from Polysciences (catalog #08691-10, lot #A774113; Warrington, PA, United States). According to manufacturer’s specification, these non-functionalized spheres contain a slight anionic charge from the presence of sulfate esters. Prior to mixing with proteins, all nanoparticles were dialyzed against 1 L of buffered solution, and the pH of PSNP solutions was confirmed after dialysis.

### Biofilm Assays

To determine the effects of the R2ab and amidase domains on biofilm formation of *S. epidermidis*, biofilm assays were performed as described previously and outlined below (Merritt et al., 2005; Billings et al., 2013). Briefly, different wells in a flat-bottomed 96-well polystyrene plate (Thermo Fisher, catalog #15041), were coated with 100 μL 0.1 mg/ml of either R2ab, amidase, or bovine serum albumin (BSA) (negative control). For protein coating, each of these proteins in gel filtration buffer (specified above) was added to a well to incubate overnight at 4°C. After incubation, the protein solution was removed by pipetting. To prepare the bacterium inoculum, *S. epidermidis* strain 1301 (available on request), a biofilm producing strain, was inoculated from a stab culture in 1 mL of Brain Heart Infusion (BHI) media at 37°C, overnight with shaking (Harris et al., 2016). To each coated well, 100 μL of fresh BHI media was added, followed by 2 μL of the overnight seed culture. The plate was allowed to incubate statically at 37°C for 72 h, allowing biofilms to form. Following the incubation and removal of the excess cells with three washings of 100 μL of PBS buffer (removed by pipetting), the biofilms were fixed with 100 μL of 100% methanol at room temperature for 10 min and stained with 100 μL 0.1% w/v crystal violet stain in water (B12525, Thermo Fisher Scientific). The biofilms were washed three times with PBS buffer as above to remove excess crystal violet stain, and 100 μL of 30% acetic acid was used to solubilize the cell bound crystal violet. Biofilm formation was monitored by measuring optical density of each well using a SpectraMax 4 plate reader at 570 nm.

### Antimicrobial Assay

To determine the antimicrobial properties of the R2ab and amidase and amidase domains, R2ab and amidase were added to cultures of *S. epidermidis* in 96 well polystyrene plates. An overnight seed culture of *S. epidermidis* strain 1301 was prepared in BHI media as described above. Then, 2 μL of this seed culture was added to 100 μL of fresh BHI media in the 96-well plate. R2ab and amidase (stock concentration of 100 μM) in gel filtration buffer were then added to the culture, followed by pipette mixing. The bacterial culture was monitored for a period of 24 h by measuring the OD_600_ every hour, at a temperature of 37°C, using a Cytation5 plate reader.

### Dynamic Light Scattering and Zeta Potential Measurements

Varying concentrations of proteins, starting from 1 μM, were added to 40 nM of nanoparticles, to a final volume of 50 μL. After nanoparticle – protein mixture was incubated at room temperature for 1 h, the samples were centrifuged at 15,000 × *g* for 10 min. The supernatant was discarded, and the sample pellet was washed three times with 10 mM sodium phosphate (pH 7.0), to remove any free protein in the solution. The pellet was then resuspended in the same buffer and the sample was transferred to a Low Volume Univette (Anton Paar). All measurements were obtained using an Anton Paar Litesizer 500 DLS at 25°C, and the data was processed using the Kalliope software.

### Circular Dichroism Spectropolarimetry

The circular dichroism (CD) measurements were carried out using a Jasco 1500 CD spectrometer at 25°C. The measurements were performed at a path length of 1 mm using a quartz cuvette. In order to determine how the secondary structure of both the proteins change in the presence of nanoparticles, all the protein solutions were made containing the same concentration of R2ab and amidase (3 μM) with increasing concentrations of nanoparticles present in the mixture. A lower concentration (0.6 μM) was used for BSA; this concentration was reduced because fewer BSA molecules are predicted to bind to PSNPs, and saturation of the surface is expected to occur at a lower concentration. Similarly, while the total number of proteins per nanoparticle monolayer changes dramatically with protein size, the total mass of protein in a monolayer remains roughly the same, justifying a lower molar concentration of BSA (Supplementary Table 1). A BSA concentration of 0.6 μM (or 0.04 mg/mL) is approximately the same in mg/mL as the 3 μM used for R2ab and Amidase (0.05 and 0.08 mg/mL, respectively). The buffer for all solutions was 10 mM sodium phosphate (pH 7.0). Between each sample measurement, the cuvette was carefully cleaned. Far-UV spectra were collected between 180 and 260 nm, with the scan rate set at 10 nm min^–1^ at a bandwidth of 1 nm using 4 s as the integration time. Spectra were smoothed using Savitzky-Golay filter set to a window size of 17. The data was analyzed, and the decomposition process was carried out using Jasco’s Spectra Manager software suite.

### Limited Proteolysis

The pattern of proteolytic digestion was tested on both R2ab and amidase in the presence and absence of polystyrene nanoparticles. In the experiments without nanoparticles, 0.5 mg/ml of proteins were incubated with 0.01 mg/ml of chymotrypsin (Amresco) (chymotrypsin: protein ratio 1:50) for 30 min at 22°C. Mixtures of proteins and nanoparticles were generated by adding PSNPs to a final (molar) nanoparticle to protein ratio of 1:190 for R2ab and 1:170 for amidase. These ratios were determined based on previous calculations of surface coverage (Wang et al., 2014). For digests containing PSNPs, chymotrypsin was added after mixing the proteins and nanoparticles, and allowing it to incubate for 1 h. The reactions were stopped by adding SDS-PAGE sample buffer (Bio-Rad) and heating the samples for 5 min at 95°C. For each experiment, a control sample of chymotrypsin was used, and a parallel sample of proteins without any chymotrypsin was analyzed and compared on 16.5% Tris–Tricine gels (Bio-Rad). The proteolytic products were visualized via silver staining due to low intensity of fragments.

## Results

### R2ab and Amidase on Surfaces Hinders Biofilm Formation but Not Bacterial Growth

**FIGURE 1:**
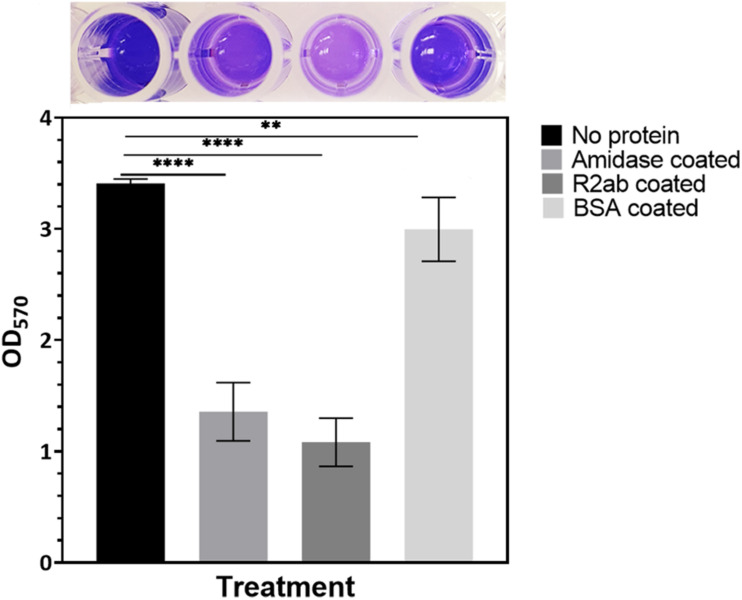
The effect of protein treatment on biofilm formation. Wells were pre-coated with either no protein (control), amidase, R2ab, or BSA, followed by growth conditions favoring biofilm formation, which was monitored by crystal violet staining and measurement of absorbance at 570 nm. The BSA-coated plate showed only a slight reduction in biofilm growth relative to no treatment, but plates treated with amidase and R2ab domains show a much larger reduction. A typical row from a stained, 96-well plate is shown above each corresponding column to display the results of crystal violet staining. The error bars for each data point represent the standard error of the mean for *N* = 8 samples. A one-way ANOVA test [*F*(3,28) = 217.2, *p* < 0.0001] with Tukey’s post hoc analysis was performed to determine the levels of significance (**p < 0.005, ****p < 0.0001).

To examine the effects of the R2ab and amidase domains on biofilm formation, we tested their effect on biofilm formation on polystyrene plates. We used *S. epidermidis* strain 1301, which is able to form dense biofilms on polystyrene surfaces. After treating the 96-well plates with either R2ab and amidase, a significant reduction in biofilm was observed, as monitored by crystal violet staining (Figure 1) (O’Toole, 2011). The absorbance at 570 nm reflects the amount of biofilm present prior to fixation and staining. As expected, the highest absorbance was obtained for the untreated wells, where cells grew unhindered in BHI media. All treatments produced a statistically significant decrease in biofilm formation, including treatment with the negative control, BSA (*p* = 0.0047). This decrease occurs because protein adsorption is a general phenomenon (Andrade and Hlady, 1986). However, in wells that had been pre-treated with either R2ab or amidase, the growth of biofilms was reduced by a substantially larger amount, a nearly three-fold reduction (*p* < 0.0001). This suggests that R2ab and amidase are far more effective than typical proteins in adsorbing to a polystyrene surface, preventing bacterial attachment and subsequent biofilm formation.

**FIGURE 2:**
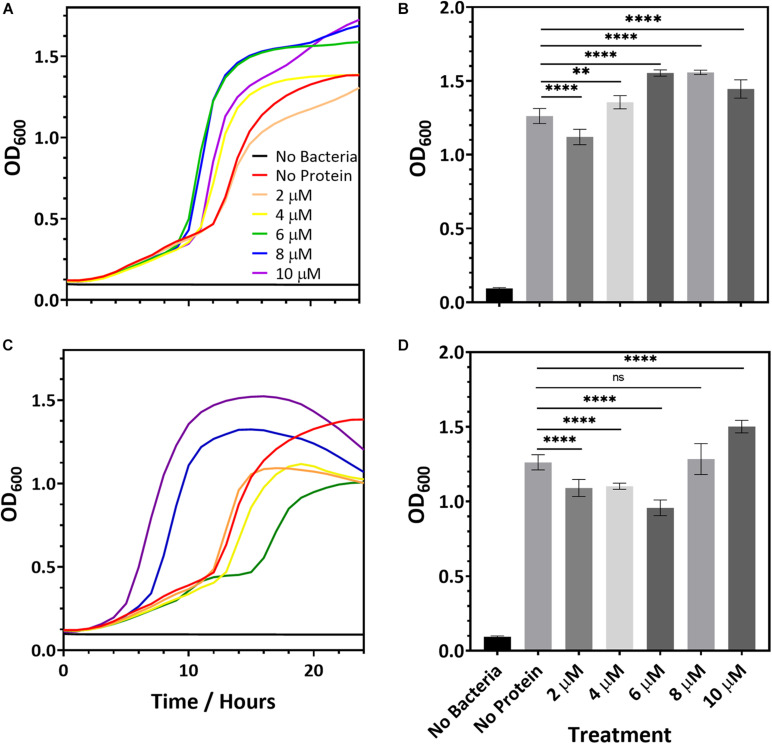
Representative microbial growth curves in the presence of R2ab **(A)** and amidase **(C)**. Growth curves are shown for *S. epidermidis* when the media was mixed with **(A,B)** R2ab or **(C,D)** amidase. Concentrations added for each protein domain are 0 μM (red), 2 μM (orange), 4 μM (yellow), 6 μM (green), 8 μM (blue), and 10 μM (purple). The black curve shows a control with no bacteria added. Panels **(B,D)** show the average and standard deviation of OD_600_ at 18 h for R2ab and amidase, respectively, for the different treatments (*N* = 8). A one-way ANOVA test [**(B)**: *F*(5, 42) = 120.4, *p* < 0.0001 and **(D)**: *F*(5, 41) = 77.51, *p* < 0.0001] with Tukey’s post hoc analysis was performed to determine the levels of significance between groups (n.s. is not significant; **p < 0.005 and ****p < 0.0001).

While R2ab and amidase may hinder biofilm formation, it is also possible that they slow bacterial growth. To determine whether this is the case, we added R2ab and amidase to the growth media of *S. epidermidis* strain 1301 and monitored the bacterial growth curves, measuring the OD_600_ at fixed intervals. Over a period of 24 h the growth curve of *S. epidermidis* was monitored in BHI media containing increasing protein concentrations for both R2ab and amidase domains. These curves were compared to curves where no proteins were added to the media (Figure 2). Some differences in growth curves were observed between cells treated with proteins and those without; however, this likely reflects small changes in the number of initial cells used to inoculate each well. Indeed, significant variability was observed for when each well reached log phase. In no case did the added R2ab or amidase lower the final OD_600_ for the cultures, and in several cases, the final cell density was increased. Moreover, as indicated by representative growth curves (Figures 2A,C), the doubling time did not appear to be affected by the addition of increasing concentrations of R2ab or amidase. At 18 h, differences between the OD_600_ values for each cultures were all statistically significant (Figures 2B,D, measured using one-way ANOVA). However, there was no apparent dose-response for treatment with either R2ab or amidase, and in many cases the treated cells grew to a higher OD_600_. These data indicate that R2ab and amidase have no antimicrobial properties that could contribute to the stunted biofilm formation observed in Figure 1.

In the pre-treatment experiments, the R2ab and amidase domains were removed from each well, leaving only the proteins that had adsorbed to the polystyrene surface. The concentration of proteins in solution if desorption occurred is therefore expected to be much less than 10 μM. However, our bacterial growth experiments show no consistent effect on cell division for concentrations of protein in solution up to 10 μM. Together, these results strongly suggest a mechanism whereby biofilm formation is blocked by adsorption of R2ab and amidase to the polystyrene surface, and not a mechanism where cell density is reduced by the presence of these domains in solution.

### R2ab and Amidase Domains Strongly Interact With Polystyrene Nanoparticles

Performing biophysical experiments of proteins on macroscopic, flat polystyrene surfaces is extremely challenging because of the low surface-volume ratio of flat surfaces and the corresponding small number of proteins adsorbed. Nanoparticles have a very high surface to volume ratio, and titration of nanoparticles into solution allows for the controlled addition of surface binding sites in an experiment. While the effects of curvature (Gagner et al., 2011; Woods et al., 2016) and surface functionalization (Dominguez-Medina et al., 2016) play a significant role in nanoparticle-surface interactions, these effects can be minimized by using proteins that are much smaller than the nanoparticles in question and by minimizing surface functionalization. To explore the structural consequences of adsorption for the R2ab and amidase domains, we examined their behavior in the presence of spherical 20 nm (nominal diameter) carboxylate functionalized polystyrene nanoparticles (PSNPs). When protein solubility conditions permitted, we also examined protein adsorption to neutral, non-functionalized PSNPs. These nanoparticles are substantially larger than either protein domain, and hundreds of copies of each protein could fit in a monolayer on each nanoparticle (138 and 150 proteins per nanoparticle for R2ab and amidase, respectively; see Supplementary Table 1) (Wang et al., 2014), suggesting that, from the perspective of each protein domain, the surface is effectively flat. PSNPs have been used extensively in protein binding measurements (Baier et al., 2011; Contado et al., 2019; Kihara et al., 2019), providing fruitful information on protein binding, corona formation, and structural changes that occur upon adsorption. While the molecular similarities between PSNP adsorption and macroscopic adsorption remain unclear, we hypothesize that the processes are related, and we set out to explore the behavior of R2ab and amidase when adsorbed to PSNP surfaces, both with and without COOH functionalization.

To examine the interactions between the R2ab and amidase domains with PSNPs, DLS profiles were measured as the protein domains were titrated into the solutions containing PSNPs. A large interaction was observed for R2ab and amidase, as monitored by the dramatic shift in hydrodynamic diameter (Supplementary Figure 1). The hydrodynamic diameter of the carboxylate PSNPs alone was found to be 42.1 ± 0.1 nm; R2ab increased this value to 3,600 ± 300 nm, and amidase-coated PSNPs had an apparent hydrodynamic diameter of 670 ± 30 nm. However, the shift for BSA was much smaller, which had a final hydrodynamic diameter of only 48.5 ± 0.3 nm. The same trend was observed for neutral, non-functionalized PSNPs (Supplementary Figure 1). No visible aggregation was observed for any of the proteins under these conditions, suggesting that dynamic association of protein domains on the PSNP surfaces was occurring, as opposed to the formation of insoluble protein-nanoparticle conjugates. The interaction occurred at very low protein concentrations, indicating that the binding between both domains and the surface was rather strong. This is similar to what was observed in a prior study of PSNP-protein interactions in bovine α-lactalbumin, which reported strong but dynamic interactions in protein adsorption, associated with increases in hydrogen-deuterium exchange rates (Engel et al., 2004). Based on the significant increase in hydrodynamic diameter measured by DLS, R2ab, and amidase interact with PSNPs to a greater degree than BSA. This is consistent with the idea that R2ab and amidase domains interact with polystyrene surfaces in a uniquely strong way.

**FIGURE 3:**
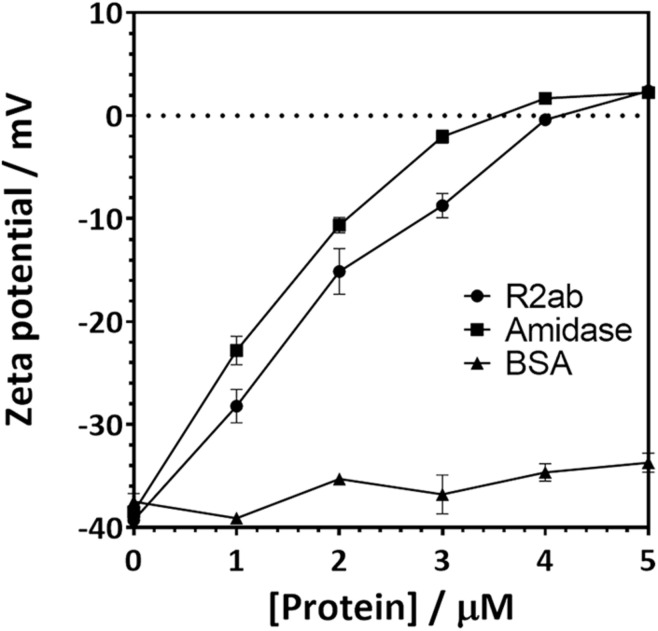
Zeta potential changes when carboxylate functionalized PSNPs are titrated with autolysin domains. The zeta potential of 20 nm (nominal diameter) carboxylate-functionalized polystyrene nanospheres increases when R2ab or amidase domains are added to solution, but very little change is seen for BSA. Error bars represent the standard deviation for *N* = 3 experiments, and lines connecting each point are added as a guide to the eye.

Zeta potential measurements for PSNPs coated with amidase and R2ab also reflect a strong interaction (Figure 3). Zeta potential measures the electric potential between at the slipping plane surrounding a surface and reflects the surface charge of a surface (Jiang et al., 2009). The zeta potential for both titrations starts at a negative zeta potential value for the bare carboxylate PSNPs, reflecting their net negative charge from carboxylic acid groups. The mV values become positive as the proteins are added in increasing concentrations. Initially, the variation of zeta potential is reasonably even; however, at higher concentrations of proteins, the zeta potential becomes fairly constant and the titration curve flattens out. The R2ab and amidase domains have theoretical isoelectric points of 9.7 and 6.8, respectively, and they are expected to decrease the net surface charge of carboxylate PSNPs as they adsorb. Assuming monolayer surface coverage, both proteins are expected to completely saturate the surface of 40 nM of PSNPs when 3–4 μM protein is present (Wang et al., 2014), and for titrations of both protein domains, the surface potential of PSNPs appears to be neutralized at this level of saturation. On the other hand, the BSA negative control does not induce a strong change in zeta potential as it is added (Figure 3), even though the degree of saturation is far greater when 4 μM is present, since far fewer molecules of BSA are able to bind in a single monolayer of a PSNP based on geometric considerations. Together, the DLS and zeta potential experiments reveal a strong interaction between amidase domains and PSNP surfaces that doesn’t appear to be present for other proteins like BSA. This is consistent with our biofilm assays on macroscopic polystyrene surfaces.

### Polystyrene Nanoparticles Alter the Secondary Structure of Autolysin Domains

**FIGURE 4:**
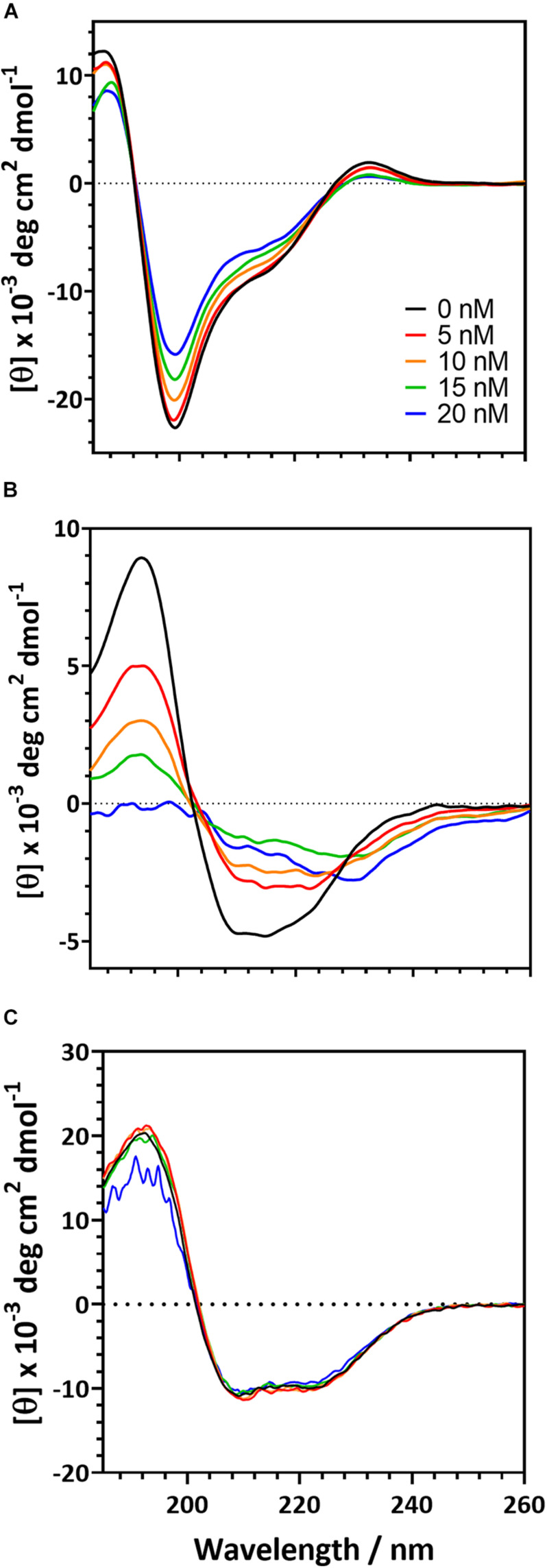
Circular dichroism spectra for the of autolysin domains and a negative control in the presence of carboxylate functionalized PSNPs. R2ab **(A)** and amidase **(B)** exhibit changes in their CD spectra when nanoparticle concentrations are increased. In the absence of nanoparticles (black curve), both domains exhibit a well-defined secondary structure. As nanoparticles are increased to 5 nM (red), 10 nM (orange), 15 nM (green), and 20 nM (blue), secondary structure changes become evident. The same changes are not observed for BSA **(C)**, whose spectrum remains fairly constant as PSNP concentration is altered.

To further understand the behavior of proteins in the presence of a polystyrene surface, we used CD to monitor changes in protein secondary structure as PSNPs were added (Figure 4). The magnitude of the CD signal decreases as PSNP concentration is increased for both the R2ab and amidase. However, the behavior of both proteins differs slightly. The R2ab domain exhibits a uniform decrease in magnitude as PSNPs are added (Figure 4A). This type of scaling behavior could result from one of two phenomena: First, if protein-PSNP conjugates were sedimenting over time, the amount of protein in solution would decrease, leading to a lower signal compared to the non-interacting reference. Fewer protein molecules would then be present in solution, which would scale the molar residue ellipticity in the presence of PSNPs. Second, it is possible that the secondary structure is changing in such a way as to reduce the CD signal, even while the total protein concentration in the optical path is remaining constant. A combination of both effects is also possible; however, we believe that secondary structure changes are the primary cause for the spectra in Figure 4. This is because no aggregation is observed in the cuvette and the spectra are stable for at least an hour. The samples were prepared having the same total protein concentration, so the scaling observed is not simply a dilution effect. In these experiments, the R2ab is in excess of the predicted nanoparticle binding sites, even at the highest nanoparticle concentration of 20 nM. Therefore, it is likely that the changes in R2ab, while uniform across all wavelengths, reflect a change in secondary structure. The behavior of the amidase domain is different, and this spectrum is not uniformly scaled as the spectrum for R2ab is. Instead, the spectral shape for amidase changes, giving rise to isodichroic points at 205 nm and potentially near 230 nm. The lower signal to noise of the 20 nM titration point for amidase likely reflects increased scattering as larger agglomerates form, similar to what was observed in the DLS experiments. This behavior also suggests a secondary structure change in the amidase domain in the presence of PSNPs, and the isodichroic points indicate that a two-state transition may be occurring. The third protein investigated, BSA, shows a very minor change compared to the other two proteins. Structural changes in BSA, if present, are very marginal, and this is consistent previous observations of BSA on carboxylate-functionalized polystyrene nanospheres (Fleischer and Payne, 2014).

These experiments were also repeated on neutral, non-functionalized PSNPs to test whether the carboxylate groups on the PSNP surface were influencing the protein structure. CD is sensitive to scattering in the far-UV range, and we found that the neutral PSNPs would aggregate and give poor signal at high protein concentrations. This made collection of CD data more challenging under these conditions. Nevertheless, trends in the CD spectra were consistent for R2ab, amidase, and BSA collected with neutral PSNPs (Supplementary Figure 2). Importantly, R2ab and amidase looked very similar on neutral PSNPs as they did on carboxylate functionalized PSNPs, whereas BSA showed very little change on both neutral and carboxylate coated surfaces. In addition, the spectral changes for R2ab and amidase were remarkably consistent with those shown in Figure 4: the R2ab spectrum exhibited a uniform scaling behavior, and the amidase spectrum became flatter in the presence of non-functionalized PSNPs. The carboxylate PSNPs used in these experiments, therefore, appear to induce similar (albeit not identical) behavior to what is observed for neutral, non-functionalized PSNPs, and this makes sense given the number density of COOH groups on the surface (see section “Discussion”).

Secondary structure analysis supports the interpretation given above (Table 1) (Greenfield and Fasman, 1969; Johnson, 1990). Upon singular value decomposition (SVD) analysis, the proteins experience quantitative changes in their secondary structure. The R2ab domain consists of primarily β-strands (Zoll et al., 2012), and this is reflected in the initial estimates of secondary structure. Similarly, the SVD analysis of the free amidase domain reflects its α/β fold (Zoll et al., 2010). Thus, in the absence of nanoparticles, both R2ab and amidase appear to be folded, with secondary structure content that agrees with their three-dimensional protein structures. This is also true for the BSA control. Upon interaction with the PSNPs, R2ab and amidase seem to lose their secondary structure, and adopt a different conformation on the PSNP surface. For R2ab, it is observed that the values for helix and turn are absent. Predictably, these values do not change due to the interaction with PSNPs, but the β-sheet content decreases. Amidase, on the other hand, consists of three well defined secondary structure components, namely, helix, sheet and turn – with helices making up for more than half of the total secondary structure of the protein. In the presence of PSNPs, all three secondary structure compositions decrease, indicating that the protein may lack regular secondary structure when bound to the nanoparticle surface. Observations in loss of secondary structure are very common and can occur due to variations in temperature (Lighezan et al., 2016) and due to interaction with surfaces (Ghosh et al., 2016). BSA, as expected, does not exhibit the same magnitude of changes observed for R2ab and amidase, and its structural decomposition is the same to within 10% for all secondary structure categories.

**TABLE 1:** Apparent secondary structure of R2ab, amidase, and BSA in the absence and presence of carboxylate PSNPs, averaged over four independent experiments.

**Protein**	**Helix (%)**	**Sheet (%)**	**Turn (%)**	**Other (%)**
**R2ab domain**				
0 nM PSNPs	0	84.6	0	15.4
20 nM PSNPs	0	67.2	0	32.7
**Amidase domain**				
0 nM PSNPs	53.2	14.8	23.7	8.3
20 nM PSNPs	44.6	11.4	19.2	24.8
**BSA domain**				
0 nM PSNPs	76.2	0	12.9	10.9
20 nM PSNPs	69.6	4.4	13.2	12.8

One must use care when performing structural analysis on proteins interacting with nanoparticle surfaces using CD. As stated above, the samples are likely a mixture of protein conformations, some of which are adsorbed and some of which are free in solution. However, SVD analysis methods were developed for pure, structurally homogeneous proteins with a well-known concentration (Johnson, 1990). Importantly, CD-based secondary structure assignment cannot be performed on mixtures of folded and unfolded proteins (Toumadje and Johnson, 1995). Therefore, these methods may not apply to rapidly interconverting mixtures of folded, unfolded, and adsorbed proteins, where the populations of each species are not known. While our analysis in Table 1 provides evidence that the secondary structure is indeed changing for R2ab and amidase, the precise percentages are not likely to be accurate in this analysis, and therefore our values for the secondary structure in the presence of PSNPs should be interpreted as an *apparent* fractional secondary structure.

Together, the changes in the CD spectral signatures, along with the secondary structure analysis, support a model where the autolysin domains interact with PSNP surface and (at least partially) unfold upon interaction. BSA, our control protein, does not appear to behave in this way, and the structural factors that make R2ab and amidase such effective polystyrene binders remain unclear. Previous work using saturation-transfer difference NMR has shown that aromatic groups can interact strongly with PSNP surfaces (Zhang et al., 2017; Zhang and Casabianca, 2018), and these are abundant in the core of these globular domains. R2ab and amidase unfolding on the PSNP surface is therefore likely mediated by these aromatic-surface interactions, and these interactions could potentially mediate the initial attachment of *S. epidermidis* bacteria during biofilm formation on polystyrene.

### Limited Proteolysis Reactions

**FIGURE 5:**
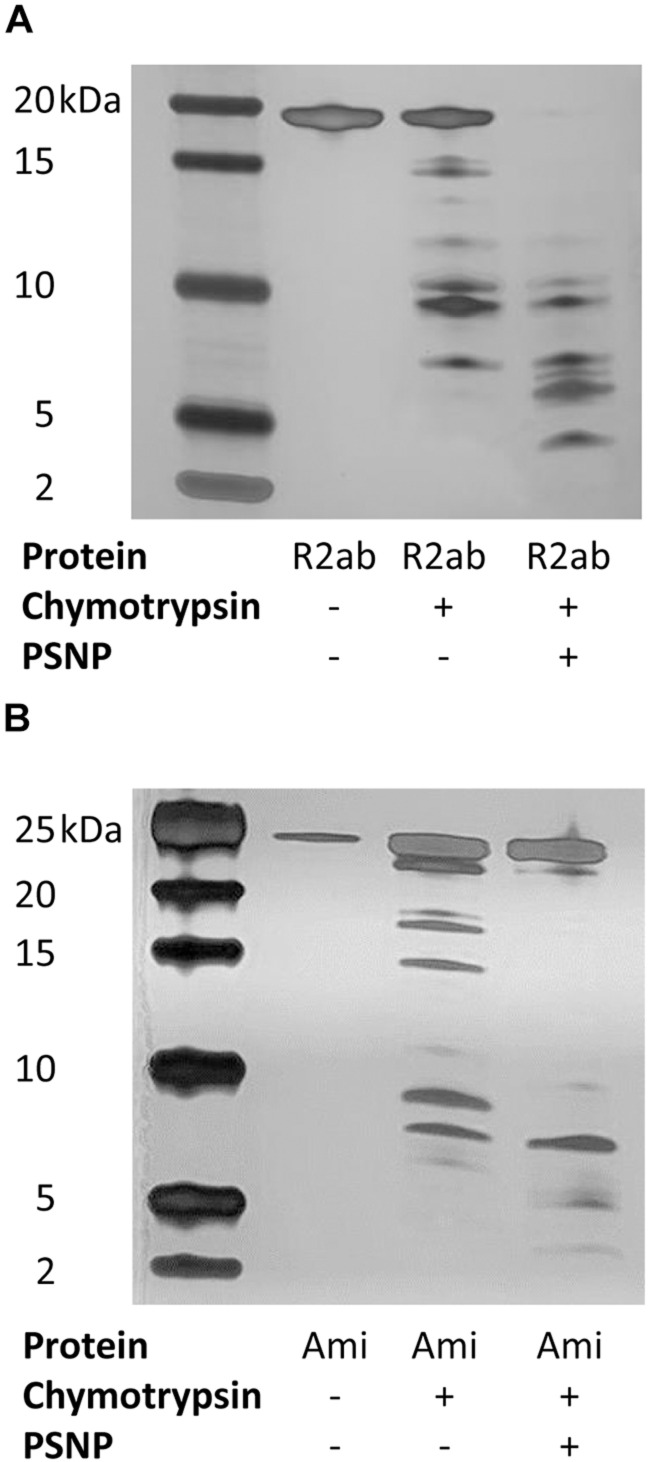
Limited proteolysis of autolysin domains. Representative silver-stained products of chymotrypsin cleavage of R2ab **(A)** and amidase [Ami, **(B)**] domains in the presence and absence of carboxylate functionalized PSNPs. The leftmost lane contains markers, and molecular weights are labeled. Limited proteolysis is more complete in the presence of PSNPs for both proteins, suggesting partial unfolding in the presence of nanoparticles.

While the spectroscopic changes we observe reflect global perturbations to protein structure, it is not clear whether any favored conformations exist when R2ab or amidase interact with the PSNP surface. To investigate this question, we performed limited proteolysis of autolysin domains in the presence of PSNPs using chymotrypsin. Because of the solubility challenges faced above with neutral PSNPs, these experiments were performed only with carboxylate functionalized PSNPs. Limited proteolysis can be used to monitor changes in exposed sites in the presence and absence of nanoparticles (Iwamoto et al., 2013; Dal Cortivo et al., 2018; Duan et al., 2019; McClain et al., 2020). If a cleavage site is protected in the presence of PSNPs, the pattern of proteolytic fragments observed on an SDS-PAGE gel will change relative to the pattern observed with no nanoparticles. Limited proteolytic reactions were compared with the uncleaved domains and with chymotrypsin to verify the nature of any ghost bands, if present. Clear differences in the cleavage patterns of both proteins were observed with and without nanoparticles (Figure 5). Due to the high sensitivity of silver staining, even less abundant populations of cleaved products were visible indicating the interaction of both R2ab and amidase with polystyrene nanoparticles. Under identical conditions, more complete cleavage was observed in the presence of PSNPs, suggesting a destabilization of these proteins when interacting with polystyrene surfaces. In addition, several low-molecular weight bands in both R2ab and amidase were stabilized. These bands likely correspond to protein fragments that have adsorbed to the PSNP surface and are partially protected from proteolysis. Chymotrypsin cleaves primarily at aromatic amino acids, which are known to favorably interact with PSNPs (Zhang and Casabianca, 2018). Therefore, significant alterations of chymotrypsin cleavage in the presence of PSNPs is anticipated and may reflect specific favorable conformations of R2ab and amidase on the nanoparticle surface.

## Discussion

When proteins encounter surfaces in solution of any type, there is a strong tendency for those proteins to adsorb to the surface. However, the similarities and differences between nanoparticle binding and macroscopic surface binding remain largely uninvestigated. Experiments have been performed to characterize binding to polystyrene nanoparticles (Kokkinopoulou et al., 2016; Contado et al., 2019), and several experiments have investigated protein adsorption to flat surfaces (Roach et al., 2006; Ngandu Mpoyi et al., 2016). In some cases, a significant influence from surface curvature is observed (Gagner et al., 2011), but in others, no effect seems to be observed. Nanoparticle surface functionalization also likely plays a significant role (Pelaz et al., 2015).

While studies of corona formation on nanoparticles abound, however, experiments comparing nanoparticle coronas with binding on macroscopic surfaces are far fewer. One reason for this is that surface functionalization on nanoparticles is often very different than what is found on macroscopic surfaces, hindering a direct comparison. Another reason is that rigorous, biophysical characterization of protein binding to flat surfaces is technically difficult: The surface to volume ratio for a flat surface is much smaller than it is for a nanoparticle, and flat surfaces are not amenable to many types of biophysical spectroscopies. For example, it would be impossible to perform the CD analysis used here on proteins adsorbed to a transparent flat surface; there is simply not enough detectable signal for such a measurement. Some methods, like attenuated total reflection Fourier transform infrared spectroscopy (Wang et al., 2006) and quartz crystal microbalance measurements (Reimhult et al., 2008) can be used to monitor proteins adsorbed to polystyrene, but these do not typically report on a protein’s global structure. Direct comparisons between nanoparticle binding and macroscopic, flat surface binding are therefore challenging, even though the soluble nature of many nanoparticles make them an attractive target for protein-surface interaction studies.

In this work, we investigated two domains of staphylococcal AtlE, a protein known to bind to polystyrene surfaces (Houston et al., 2011). We sought to compare protein-surface binding using three scenarios: (1) the flat surface of polystyrene well plates, (2) the curved surface of neutral, non-functionalized PSNPs, and (3) the curved surface of carboxylate-functionalized anionic PSNPs. Although the PSNPs are curved, the radius of curvature is far larger than the characteristic radius of gyration for amidase and R2ab (16.3 and 17.7 Å, respectively) (Wang et al., 2014). This suggests that proteins encounter an effectively flat surface when adsorbing to these PSNPs. The major difference between the surfaces is the functionalization: With a zeta potential of −40 mV, the carboxylate functionalized PSNPs used here have a surface density of approximately 60 COOH groups per particle. This number is zero on the neutral, non-functionalized PSNPs and the polystyrene plates. The number of COOH groups is significant, and their presence makes carboxylate-PSNPs much more soluble than they would be otherwise; however, these groups could potentially interfere with protein adsorption. Our rationale for including carboxylate functionalized PSNPs was based on geometric considerations: Specifically, R2ab is expected to occlude approximately 980 Å^2^ of surface based on its radius of gyration, and amidase is expected to occlude approximately 840 Å^2^. For comparison, on average one carboxylic acid group is found for each 4,300 Å^2^ on the carboxylate-PSNP surface (using the manufacturer’s lot-specific certificate of analysis). Thus, there will be far fewer acidic groups on the surface than directly adsorbed proteins. Moreover, other ionic species in solution will also be drawn to the PSNP surface, and this will tend to screen electrostatic interactions through a Debye-Hückel effect, potentially making protein adsorption less dependent on the PSNP functionalization. Our goal in this work was to establish what similarities (if any) exist between AtlE’s role in surface binding and biofilm formation and its ability to interact with PSNPs. Including both anionic, carboxylate functionalized PSNPs and neutral, non-functionalized PSNPs allowed us to control for the presence of COOH groups, even though the neutral nanoparticles suffered from significant problems with solubility at high protein concentrations.

While the importance of AtlE in polystyrene surface binding has been established (Heilmann et al., 1997; Houston et al., 2011), surprisingly, we find that both domains bind tightly to PSNPs as well. The R2ab and amidase domains, when adsorbed to a polystyrene surface, significantly hinder *S. epidermidis* biofilm formation in a way that other proteins, like serum albumin, cannot (Figure 1). This is likely because the R2ab and amidase domains, when present, occlude the polystyrene surface in the well plate, preventing the bacteria’s own R2ab and amidase from binding. In other words, pre-coating the polystyrene with recombinant R2ab and amidase prevents the bacteria-attached AtlE and other components, such as polysaccharide intercellular adhesin (PIA) or extracellular DNA, from encountering any free polystyrene surface area. This demonstrates that these domains interact directly with the surface and out-compete R2ab and amidase from the bacteria, reducing the bacteria’s ability to attach to surfaces. The interaction with the surface is sufficiently strong enough to withstand multiple washes and overnight incubation, and the bacterial growth experiments demonstrate that the effect is attributable to surface attachment alone as opposed to an antimicrobial effect. To our knowledge, this study is the first to show a direct, competitive effect between AtlE domains for surface binding; moreover, it demonstrates that both domains bind polystyrene surfaces strongly enough to hinder bacterial attachment. Thus, when a polystyrene surface is precoated with the autolysin domains, the surface is masked by the proteins, and the microbes cannot recognize the polystyrene well plate as a surface for colonization.

Interestingly, the R2ab and amidase domains also bind PSNPs very tightly. The average particle diameter for this lot of PSNPs is 28 ± 6 nm by TEM. Based on the size of the proteins and surface area of the nanoparticles, geometric considerations predict that a well-packed protein monolayer on a 28 nm PSNP contains approximately 250 R2ab molecules and 300 amidase molecules (Wang et al., 2014). The experiments performed in this work were done at conditions at or below this stoichiometry, and zeta potential measurements appeared to stabilize by this point, suggesting that the interaction is very strong. While zeta potential is less informative for neutral, non-functionalized PSNPs, both neutral and carboxylate PSNPs show a significant increase in hydrodynamic diameter by DLS, which also indicates a strong interaction. At this point, it remains unclear whether this interaction with PSNPs is mediated by similar molecular interactions as are seen in the flat polystyrene surfaces. Nevertheless, a strong interaction is observed between the AtlE domains and all three types of surfaces: carboxylate PSNPs, neutral PSNPs, and flat polystyrene. The common theme for all of these is polystyrene, and therefore the modes of interaction are likely to be similar.

Both R2ab and amidase are proteins with a highly ordered structure (Zoll et al., 2010, 2012). This is evident from the secondary structure, as monitored by CD, in the absence of PSNPs. In this study, we observed significant conformational changes of both R2ab and amidase upon interaction with PSNPs. Importantly, key secondary structure elements are lost when the proteins interact with nanoparticles, and an increase in coil is observed. Such measurements are difficult to interpret quantitatively, since the solution in the presence of PSNPs likely contains a mixture of free and bound states, where the nanoparticle-bound states are partially denatured. However, adsorption to a polystyrene surface appears to disrupt the structure of both proteins. Once again, a similar trend is seen regardless of the surface functionalization, as neutral PSNPs exhibit the same qualitative changes as observed for the more soluble carboxylate functionalized PSNPs. Quantifying the changes for the neutral PSNPs is difficult because of scattering and protein solubility of these nanoparticles, and slight differences in the CD spectra may reflect small changes in how R2ab and amidase bind to neutral polystyrene; nevertheless, the similarities between the CD spectra are striking (Figure 4 vs. Supplementary Figure 2). Structural perturbations in PSNP-adsorbed proteins have been observed before: Engel et al. (2002) observed near-complete unfolding of α-lacalbumin when adsorbed to polystyrene spheres, and Salvati et al. (2013) observed less extensive structural changes in adsorbed transferrin. There appears to be a range of behaviors that are likely influenced not only by the PSNP surface itself, but also the protein structure and stability (Woods et al., 2016; Perera et al., 2019). The increased disorder observed for the AtlE domains likely exposes hydrophobic and aromatic groups, which can interact favorably with the PSNP surface (Zhang et al., 2017; Zhang and Casabianca, 2018).

The destabilization of the R2ab and amidase domains is further highlighted by enhanced limited proteolysis in the presence of carboxylate PSNPs. Limited proteolysis is an irreversible process which can be employed to obtain structural information on protein interactions, and it has been applied to study the interactions between folded proteins and nanoparticles (Iwamoto et al., 2013; Dal Cortivo et al., 2018; Duan et al., 2019). Limited proteolysis is a label-free method, employing a short digestion step where proteases cleave at specific accessible residues present in the folded protein (Brownridge and Beynon, 2011). If unfolding occurs in the presence of nanoparticles, the number of solvent accessible amino acids would change, potentially altering the number of proteolytic cleavage sites. Indeed, this is what is observed for R2ab and amidase: proteolysis is accelerated in the presence of PSNPs, and when nanoparticle fragments are present, fragments of smaller size are generated during a limited chymotrypsin digest (Figure 5). This result not only confirms the structural changes observed by CD, but the absence of any large fragments in PSNP digest suggests the protein is uniformly destabilized over the entire sequence. In other words, no stable subdomains appear to be protected from proteolysis when PSNPs are present.

## Conclusion

In this study we present several approaches for examining the interaction between the domains of AtlE and polystyrene surfaces. While AtlE is known to be important in S. *epidermidis* attachment to biofilm surfaces, we have demonstrated that both the amidase and R2ab domains are capable of binding polystyrene tightly and can significantly reduce biofilm formation when applied to a polystyrene surface. The mechanism appears to be that these domains pre-coat the surface, preventing binding from bacteria-associated AtlE. Importantly, this effect is much reduced for serum albumin, a negative control. To study the potential structural and biophysical consequences of protein adsorption, we employed anionic, carboxylate functionalized and neutral, non-functionalized PSNPs, which increase the surface-to-volume ratio and allow for spectroscopic and proteolytic characterization of the corona of adsorbed protein. Both AtlE domains also bind tightly to PSNPs, and structural changes are observed that suggest the proteins partially unfold upon binding to polystyrene surfaces. Importantly, the structural changes detected by CD are similar for both carboxylate and non-functionalized PSNPs, suggesting that the modes of binding for AtlE domains may also be similar for different surface types. AtlE provides a useful and biomedically relevant test case for studying surface binding because of its involvement in pathogenic biofilms and its strong interaction with polystyrene. However, additional work is needed to investigate other proteins, focusing on adsorption mechanisms to nanoparticle surfaces and to chemically related macroscopic surfaces. If general similarities are observed, then nanoparticles may prove to be a useful tool for studying how proteins interact with many different types of surfaces.

## Data Availability Statement

The original contributions presented in the study are included in the article/Supplementary Material, further inquiries can be directed to the corresponding author.

## Author Contributions

RS, YP, and NF conceived and designed the experiments. RS, EC, RY, JP, and NF performed the experiments. RS, EC, YP, and NF wrote and edited the manuscript. All authors contributed to the article and approved the submitted version.

## Conflict of Interest

The authors declare that the research was conducted in the absence of any commercial or financial relationships that could be construed as a potential conflict of interest.
