# Supplementary material for: Understanding How Staphylococcal Autolysin Domains Interact With Polystyrene Surfaces
Source: Front Microbiol. 2021 May 19;12:658373. doi: 10.3389/fmicb.2021.658373 (PMC8170090; doi:10.3389/fmicb.2021.658373)
Supplement: Supplementary file 1 [file Data_Sheet_1.PDF]

Supporting Information for:

Understanding How Staphylococcal Autolysin Domains Interact with Polystyrene Nanoparticle Surfaces

Radha P. Somarathne,[1] Emily R. Chappell,[1] Y. Randika Perera,[2] Rahul Yadav,[1] Joo Youn Park,[3] and Nicholas C. Fitzkee[1]*

[1] Department of Chemistry, Mississippi State University, Mississippi State, MS 39762 USA
[2] Department of Biochemistry, Chemistry, and Center for Structural Biology, Vanderbilt University, Nashville, TN 37240, USA
[3] Department of Comparative Biomedical Sciences, College of Veterinary Medicine, Mississippi State University, Mississippi State, MS 39762 USA
* To whom correspondence should be addressed
Email nfitzkee@chemistry.msstate.edu
ORCID: 0000-0002-8993-2140
Fax: (662) 325-1618

Table S1. Predicted binding stoichiometries and mass coverage, assuming a folded, densely packed monolayer of protein on a 28 nm diameter spherical nanoparticle (NP). Calculated according to Wang, et al. 2014, and including bovine carbonic anhydrase (BCA) and bovine serum albumin (BSA).

Protein	Molecular Weight (kDa)	PDB ID*	Radius of Gyration (R_G, Å)	Predicted # of Proteins in Monolayer per NP	Mass of Protein in Monolayer per NP (ag)
GB3	6.2	2OED	10.5	710	7.3
Ubiquitin	8.6	1UBQ	12.0	540	7.8
R2ab	17	4EPC	17.7	250	7.0
Amidase	24	3LAT	16.2	300	12
BCA	29	1V9E	17.2	260	13
BSA	66	3V03	25.2	120	14

* When multiple chains were present, all structural calculations used chain ID A.

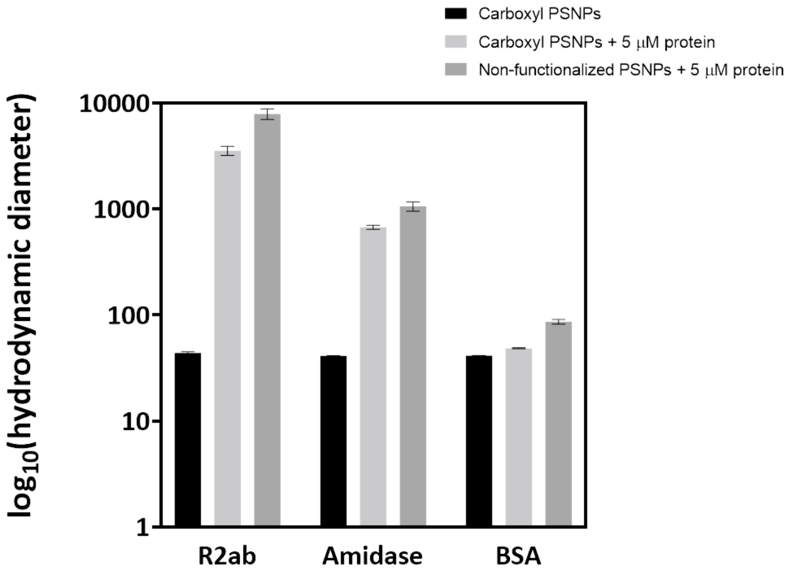

Figure S1. Hydrodynamic diameter changes measured by dynamic light scattering (DLS) when both carboxylate functionalized and non-functionalized PSNPs are mixed with 5 μM of R2ab, Amidase, or BSA. The hydrodynamic diameter increases significantly when R2ab and amidase are added to the solution, but only a marginal increase is observed when BSA is added. Error bars represent the standard deviation for three experiments.

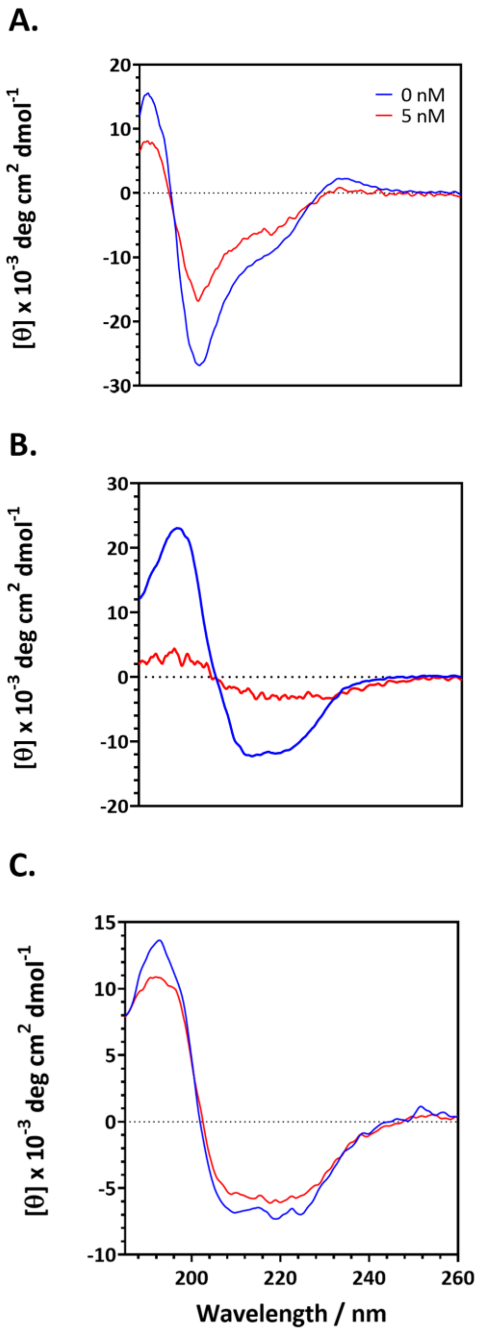

Figure S2. Secondary structure changes measured by circular dichroism (CD) when non-functionalized (neutral) PSNPs are mixed with 0.05 mg/ml of R2ab (A), 0.08 mg/ml of Amidase (B), and 0.04 mg/ml of BSA (C). The secondary structure changes when R2ab and amidase are added to the solution, but only a slight change is observed when BSA is added. This is consistent with observations for carboxylate functionalized nanoparticles.

Reference

1. Wang, Ailin, Karthikeshwar Vangala, Tam Vo, Dongmao Zhang, and Nicholas C. Fitzkee. 2014. "A Three-Step Model for Protein–Gold Nanoparticle Adsorption." *The Journal of Physical Chemistry C* 118 (15): 8134–42. https://doi.org/10.1021/jp411543y
